# The Prostate cancer–Exercise and Metformin randomised controlled feasibility Trial (Pre-EMpT) in men following active surveillance, radical prostatectomy and radiotherapy

**DOI:** 10.1186/s40814-025-01654-0

**Published:** 2025-05-24

**Authors:** Luke A. Robles, Lucy McGeagh, Jonathan Aning, Amit Bahl, Amarnath Challapalli, Anthony Koupparis, Raj Persad, Edward Rowe, Constance Shiridzinomwa, Richard M. Martin, J. Athene Lane

**Affiliations:** 1 NIHR Bristol Biomedical Research Centre, University Hospitals Bristol and Weston NHS Foundation Trust and University of Bristol, Bristol, UK; 2 Nuffield Department of Primary Care Health Sciences, University of Oxford, Radcliffe Observatory Quarter, Woodstock Road, Oxford, OX2 6GG UK; 3 Oxford Institute of Applied Health Research, Oxford Brookes University, Jack Straws Lane, Marston, Oxford OX3 0 FL UK; 4 Bristol Urology Institute, North Bristol NHS Trust, Southmead Hospital, Southmead Road, Westbury-On-Trym, Bristol, BS10 5 NB UK; 5 Population Health Sciences, Bristol Medical School, University of Bristol, Bristol, BS8 2PS UK; 6 Bristol Haematology and Oncology Centre, University Hospitals Bristol and Weston NHS Foundation Trust, Horfield Road, Bristol, BS2 8ED UK; 7 Clinical Research Centre, North Bristol NHS Trust, Southmead Hospital, Southmead Road, Westbury-On-Trym, Bristol, BS10 5NB UK

**Keywords:** Prostate cancer, Physical activity, Metformin, Feasibility, Factorial

## Abstract

**Background:**

Moderate to vigorous physical activity and metformin are associated in epidemiological studies with reduced biochemical recurrence and mortality in men with prostate cancer. This study assessed the feasibility of a home-based physical activity and/or metformin intervention in men with non-metastatic prostate cancer following radical treatment (surgery or radiotherapy) or active surveillance.

**Methods:**

A 2 × 2 factorial design randomised men into one of four groups for 6 months: (1) physical activity (defined as brisk walking ≥ 30 min for ≥ 5 days per week, aiming for ≥ 10,000 steps a day); (2) metformin (one 500 mg slow-release tablet daily); (3) physical activity and metformin; and (4) control. Men were recruited from a single tertiary referral centre in the South West of England, UK, (September 2018–March 2020 which terminated slightly early due to the COVID-19 pandemic). Co-primary outcomes were rates of randomisation and adherence which was defined as men brisk walking ≥30 minutes on at least 5 days with 10,000 steps daily (measured over one week 6-months after randomisation) with ≥ 60% adherence to metformin between 3- and 6 months post-randomisation using returned pill count. Secondary outcomes included self-reported adverse events and physical activity, feasibility of wearing activity monitors and questionnaire completion.

**Results:**

In total, 295 men were eligible and 110 were randomised (37.3%, 95% confidence interval [CI] 31.8 to 43.1). Adherence to the physical activity and metformin interventions was 46.9% (95% CI 32.5 to 62.0) and 47.1% (95% CI 32.9 to 61.5) respectively. Adherence was > 60% for both the physical activity and metformin interventions on a complete case basis. Adverse events were infrequent (*n* = 7) across randomised groups. Completion of self-reported measures of physical activity, urinary incontinence, sexual function, quality of life and stages of change was over 80%. Step counts were not higher in men wearing activity monitors that alerted them about their step counts and sedentary behaviour. Retention over 6 months was 91.3% (95% CI 84.2 to 96.0). Follow up and intervention prompts were impacted by the pandemic.

**Conclusion:**

Home-based physical activity and metformin interventions show some promise for men with prostate cancer following radical treatment or active surveillance.

**Trial registration:**

ISRCTN, ISRCTN13543667. Registered 2 August 2018, https://www.isrctn.com/ISRCTN13543667

**Supplementary Information:**

The online version contains supplementary material available at 10.1186/s40814-025-01654-0.

## Key messages regarding feasibility

- What uncertainties existed regarding the feasibility?

There is limited evidence on the effects of physical activity and metformin on prostate cancer progression. This study assessed randomisation and adherence rates of a home-based physical activity and metformin intervention in men receiving radical treatment or active surveillance.

- What are the key feasibility findings?

This study demonstrates the feasibility of a factorial randomised controlled trial design in this population. An acceptable number of men were randomised to one or combined interventions (i.e. brisk walking and metformin). Adherence to both interventions was below our pre-defined criteria to potential progression to a definitive trial. However, the trial showed good retention of men, high completion rates of questionnaires (secondary outcomes), and few adverse events.

- What are the implications of the feasibility findings for the design of the main study?

Motivational reminders in the physical activity and metformin interventions were not sent to men following the COVID-19 pandemic, which may have impacted on intervention adherence. In addition, approximately 30% of men in the brisk walking and metformin interventions had missing outcome data, which meant that adherence could not be calculated for these men. Future trials should consider prompting men to assist adherence and monitor collection of outcome measures throughout the intervention period.

## Background

Prostate cancer is the second most common cause of UK male cancer death after lung cancer. In 2016, over 11,500 men died as a result of the disease in the UK [1]. Ten year disease-specific survival rates are very high (99%) in men with screen-detected localised prostate cancer, irrespective of whether they receive conservative or radical treatment [2] with survival rates slightly lower (95%) for men with locally advanced prostate cancer at ≥ 5 years [3]. However, a third of men have biochemical recurrence [4] and men treated with radical prostatectomy or radiotherapy can experience serious side effects, including urinary incontinence and sexual dysfunction, and reduced quality of life [5].

Observational studies suggest that moderate to vigorous physical activity reduces the risk of biochemical recurrence and mortality from prostate cancer [6, 7]. Emergent evidence from randomised controlled trials (RCTs) indicate that vigorous physical activity can reduce markers of biochemical progression of prostate cancer (i.e. prostate-specific antigen levels), as well as prostate cancer cell growth [8]. In addition, physical activity RCTs have shown improvements in several outcomes associated with treatment in men with non-metastatic prostate cancer, such as cardiovascular risk, quality of life, reduced fatigue and incontinence [9–11]. Many physical activity interventions in men with prostate cancer required supervised training and/or access to exercise facilities [12]. Home-based physical activity interventions potentially have greater sustainability by overcoming barriers to accessing exercise facilities and reducing costs. However, evidence of unsupervised home-based physical activity interventions in men with prostate cancer is limited, particularly in men undergoing radiotherapy and active surveillance.

Metformin, a first line medication in the glycaemic control of type 2 diabetes [13], has been inversely associated with risk of prostate and other cancer incidence, biochemical recurrence and mortality [14–16]. Existing RCTs of metformin have focused on men with high-risk or metastatic prostate cancer undergoing androgen deprivation therapy [17, 18]. Metformin is a safe and cost-effective medication. However, it is unclear whether men with non-metastatic prostate cancer, which are the majority of cancers detected through prostate cancer screening, would adhere to taking metformin as a potential cancer preventive therapy.

This study aimed to assess the feasibility of an RCT of home-based physical activity and metformin intervention in men with localised and locally advanced prostate cancer, and their adherence to these interventions at 6 months post-randomisation.

## Materials and methods

This study is reported in accordance with the CONSORT checklist (Additional file 1). The trial protocol has been published [19].

### Trial design

Pre-EMpT is a single centre 2 × 2 factorial randomised controlled open-label feasibility trial. Ethical approval was obtained in April 2018 (18/WA/0067).

### Participants

Participants were identified at a single tertiary referral centre in the South West of England, UK, between September 2018 and March 2020. Eligible participants included men aged ≥ 18 years, diagnosed with localised or locally advanced prostate cancer, due to receive either radical treatment (radical prostatectomy or radiotherapy) or active surveillance within two local NHS trusts, able to consent for themselves, and able to speak and read English. Exclusion criteria were: the inability to provide consent, unavailable for follow-ups, currently taking metformin or insulin, co-morbidities preventing participation in the metformin intervention, use of a mobility aid (except a walking stick) preventing participation in physical activity intervention.

### Randomisation

Participating men were allocated to one of four interventions: (i) physical activity; (ii) metformin; (iii) physical activity and metformin; and (iv) a control group of usual care. A computer-generated random allocation sequence was produced by a member of the research team, who was not involved in recruitment, data collection or analysis of the study. The allocation sequence used block random allocation (1:1:1:1 ratio) and was stratified by prostate cancer treatment (i.e. radical surgery, radical radiotherapy, or active surveillance) aiming to balance treatments across randomised groups. The allocation sequence was imported and concealed using Research Electronic Data Capture (REDCap) software [20]. Men were consented and randomly assigned to the interventions by the research nurse.

Three consents were obtained in this study. Consent 1 (randomisation consent) was obtained verbally over the telephone by the research nurse who randomised men. Metformin prescriptions could, then, be prepared in advance for the first clinic visit. Consent 2 (written informed consent) was obtained at the first clinic visit before men started any study procedures. Consent 3 (interview consent) was provided by men who were invited to be interviewed and was not contingent on other aspects of follow up. Investigators, participants or researchers were not blind to allocated interventions.

### Interventions

#### Physical activity and metformin interventions

Men in the physical activity intervention were asked to walk briskly for 30 min, 5 days a week, for 6 months, in addition to their normal level of physical activity. They were also asked to aim to walk 10,000 steps over the course of each day. Men in the metformin intervention were asked to take one 500 mg slow-release metformin tablet daily with food for 6 months. Men in the physical activity and metformin intervention were asked to perform the brisk walking and metformin interventions concurrently. All men in the intervention groups were given a pedometer (Yamax Digi-walker SW200 or CW600) and asked to report their daily step counts and minutes of brisk walking (physical activity intervention groups) over a 1-week interval at baseline and each follow-up (i.e. 3, 6 and 12 months) using a paper monitoring form. Men in the metformin interventions were asked to bring any unused metformin tablets to their 3- and 6-month research clinic visits for tablet counts. They were also asked to report their daily tablet adherence (yes/no) on the paper monitoring form at baseline and 3 to 6 months follow-ups.

#### Control group

Men in the control group continued their normal levels of physical activity. They were given a pedometer and asked to report their daily step counts over a 1-week interval at baseline and each follow-up (i.e. 3, 6 and 12 months).

#### Activity monitors as motivational tools

Men in the physical activity interventions were offered a wrist-worn activity monitor (Garmin Vivofit 4 monitor) as a motivational tool, thus alerting men when they had reached their walking goal (i.e. 10,000 steps a day) and when they were sedentary for an hour. Men wore the pedometer and activity monitors (if they consented) for 6 months. Step counts reported using the pedometer were used to measure adherence to the physical activity intervention for the primary outcome. Step count data were also reported using the activity monitors to assess their impact on physical activity. Other physical activity data collected by the monitor were not used in this study.

#### Motivational reminders

All intervention group men were provided with motivational messages through telephone, text or email, depending on their preference at 1, 4, 8, 14 and 20 weeks to encourage adherence and remind them to complete and return their outcome measures. Men in the control group did not receive motivational messages [19].

### Baseline and follow up clinics

At baseline, all men were provided with verbal and written instructions at a research nurse clinic following written informed consent. The research nurse recorded men’s height, weight, took non-fasting blood samples, and asked men to complete the baseline questionnaire. All men were provided with a pedometer and activity monitor (where applicable) and men in the metformin interventions were given their 3-month supply of metformin. At 3 months post-randomisation, telephone clinic appointments were offered to men in the brisk walking and control interventions if in-person clinic visits were not convenient. Men in the metformin interventions attended research clinics in person and returned any unused metformin medication for tablet counts by the research nurse. These men had their estimated glomerular filtration rate (eGFR) checked (normal age-related range > 60 mL/min/1.73^2^) before being given a further 3-month supply of metformin. At 6-months post-randomisation, all men attended in-person clinic visits for non-fasting blood samples and weight measurements.

Questionnaires and activity monitoring forms were posted to men 2-weeks before their research clinic visits at 3, 6 and 12-months post-randomisation. Men in the radical prostatectomy group completed an additional questionnaire measuring urinary symptoms at 7 weeks post-randomisation. Men in the radiotherapy group completed additional questionnaires measuring fatigue and quality of life at 4.5 months post-randomisation (Supplementary Table S1, Additional file 2). They were provided with pre-paid envelopes to return them to the research team.

#### COVID-19 amendments

In March 2020, recruitment was terminated early and data collection was halted at the study site due to the COVID-19 pandemic. Between March and August 2020, research-nurse led clinics were done remotely at 3- and 6 months post-randomisation. We were unable to send motivational reminders at 1, 4, 8, 14 and 20 weeks for 1.3% (*n* = 1), 5.3% (*n* = 4), 11.8% (*n* = 9), 18.4% (*n* = 14), 25.0% (*n* = 19) of men, respectively, receiving the interventions. Men in all intervention groups were also asked to self-report their weight during follow up if possible. Men in the physical activity interventions were advised to continue brisk walking if possible and men with at least 2-weeks of brisk walking to complete were provided with home-based step-based exercises. Men due their 3-month follow-up between March and May 2020 without eGFRs (*n* = 9, 17.6%) were not prescribed a further 3-month supply of metformin. They were asked to self-report their tablet counts over the telephone and to dispose of any unused tablets at their local pharmacy. Men unable to attend their 6-month follow-ups in-person were asked to attend for blood sample collections once lockdown restrictions eased. Data collection, including blood samples, questionnaires and monitoring forms, recommenced in September 2020 which resulted in some follow up time points being delayed. However, data were still collected from men with delayed follow-ups to assess feasibility outcomes.

### Primary outcomes

The co-primary outcomes were randomisation rate (randomised/eligible) and intervention adherence at 6 months post-randomisation (end of intervention). Physical activity adherence was defined as brisk walking for ≥ 30 min on ≥ 5 times a week and achieving ≥ 10,000 steps a day measured over a 1-week interval at 6 months post-randomisation using the pedometer. Metformin adherence for the primary outcome was defined as the difference in tablet counts administered and returned at clinic visits by the number of days between clinic visits. Men also self-reported taking metformin over a 1-week interval at 6 months post-randomisation as a secondary measure of adherence. The pre-specified criteria for potential progression to a definitive trial were (1) a randomisation rate of ≥ 20% of eligible men; (2) ≥ 50% of men brisk walking for ≥ 30 min on ≥ 5 days a week; and (3) men taking metformin tablets ≥ 60% of the time between 3- and 6 months post-randomisation.

### Secondary outcomes

Secondary outcomes at 6 months post-randomisation included (1) intervention tolerability (reported adverse events and qualitative interviews); (2) trial retention; (3) feasibility of assessing physical activity; (4) the number of men consenting to wear the activity monitors at baseline; (5) effect of the activity trackers on physical activity (number of pedometer step counts for men with and without the activity monitor); (6) questionnaire completion rates (number completed compared to number returned); (7) collection of smoking, alcohol, body height and weight data to calculate body mass index (BMI). The feasibility of measuring biomarkers of prostate cancer progression and the qualitative findings will be published separately.

### Patient reported outcomes

Physical activity levels were measured using the Godin-Shepard Leisure-time Physical Activity Questionnaire (GSLTPAQ) [21]. Urinary symptoms were assessed by the International Continence Scale—Short form (ICSmaleSF) [22], which measures voiding and incontinence symptoms, frequency during the day and at night (nocturia), and quality of life. Men’s beliefs about their physical activity were measured using an adapted Stages of Change questionnaire [23] and the Theory of Planned Behaviour questionnaire [24]. Men’s function and bother after prostate cancer treatment was measured using the Expanded Prostate cancer Index Composite-26 (EPIC-26) [25]. Prostate cancer-related fatigue was measured using the Functional Assessment of Chronic Illness Therapy-Fatigue (FACIT-F) [26]. Quality of life was measured by the EQ-5D 5 level version (EQ-5D-5L) [27] and FACT-Prostate subscale (FACT-P) [28]. Psychological factors, included mood states and positive life changes from being diagnosed with prostate cancer, were measured using the Profile of Moods State (POMS) [29] and the Benefit Finding Scale [30].

### Sample size

The sample size calculation was based on the precision (width of the 95% confidence interval, CI) of randomising 20.0% of 610 eligible men receiving the main prostate cancer treatments annually at the study site. This equated to 122 participants with 54 participants of 270 (20.0%; 95% CI 15.4% to 25.3%) who received active surveillance, 40 of 200 (20.0%; 95% CI 14.7% to 26.2%) who received surgery, and 28 of 140 (20.0%; 95% CI 14.0% to 27.6%) who received radiotherapy. As a feasibility study, it was not powered to determine differences in outcomes between randomised intervention groups.

### Statistical analysis

All statistical analyses were performed using STATA version 17. Data were analysed using the intention-to-treat (ITT) principle; eligible men were analysed according to their randomised intervention allocation. Distributions of continuous variables were checked graphically. Normally distributed continuous data are presented as mean and standard deviation (SD) with median and interquartile range (IQR) presented for non-normally distributed data. Categorical data are presented as number, percentage, and 95% confidence interval. Step counts (pedometer data) and duration (in minutes) in men undergoing the physical activity interventions (± metformin) were combined at 6 months post-randomisation to dichotomise men into an adherent (≥ 30 min on ≥ 5 days a week and ≥ 10,000 steps reported over a 1-week interval) and non-adherent group (< 30 min on < 5 days a week and < 10,000 steps). Tablet counts from the metformin interventions (± brisk walking) were combined at 6 months post-randomisation. The proportion of men adhering to the metformin intervention was calculated as the number of tablets counts administered and returned at clinic visits divided by the number of days between clinic visits. Men were considered adherent to the metformin intervention if they had taken ≥ 60% of their prescription between clinic visits. A secondary measure of metformin adherence used data from men reporting taking their tablets in a 1-week interval at 6 months post-randomisation. Missing data were not imputed but the proportion of missing data for each categorical outcome are reported.

### Data availability statement

The data generated in this study are available upon request from the corresponding author.

## Results

### Randomisation and retention

**Fig. 1:**
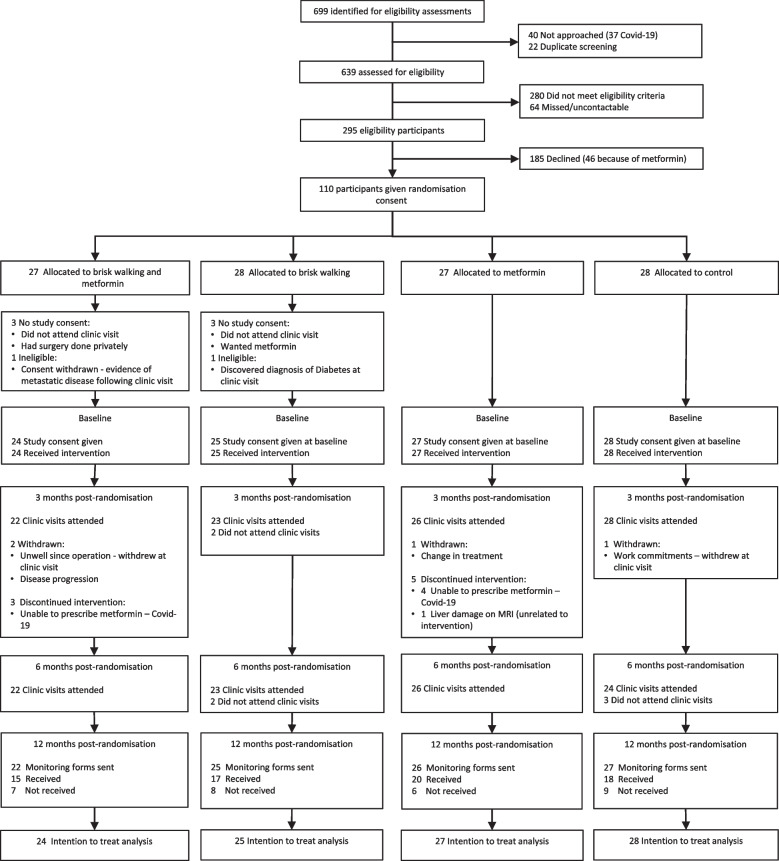
Flow chart of participants

Recruitment started in September 2018 and closed slightly early in March 2020 due to the COVID-19 pandemic. A total of 639 men were assessed for eligibility. Of the 295 eligible and approached men, 110 consented to be randomised (randomisation rate = 37.3%, 95% CI 31.8 to 43.1) (Fig. 1) and 35% (104/295, 95% CI 30.0 to 41.0) were recruited as 6 men did not provide written consent and had missing data thereafter. Follow-ups were conducted between November 2018 and May 2021. We retained 91.3% (95/104, 95% CI 84.2 to 96.0) of men randomised to the interventions at 6 months post-randomisation.

### Participant characteristics at baseline

Men had a mean age of 66.7 years (SD = 7.1), 91.3% were white ethnicity, 78.8% were married, and had a mean BMI of 28.1 kg/m^2^ (SD = 6.0). The median PSA level was 7.8 ng/ml (IQR = 5.8 to 12.7), 46.2% were undergoing active surveillance, 44.2% received surgery, and 9.6% received radiotherapy (Table 1).

**Table 1:** Participant characteristics at baseline

	Interventions									
	Brisk walking and metformin (*n* = 24)		Brisk walking (*n* = 25)		Metformin (*n* = 27)		Control (*n* = 28)		Total^a^ (*n* = 104)	
	*N*	%	*N*	%	*N*	%	*N*	%	*N*	%
Age, mean (SD)	67.4	(6.9)	64.9	(7.8)	66.6	(7.9)	67.7	(5.8)	66.7	(7.1)
Ethnicity										
White	22	91.7	20	80.0	26	96.3	27	96.4	95	91.3
Other	2	8.3	5	20.0	1	3.7	1	3.6	9	8.7
Highest level of education										
Standard education or less	10	41.7	8	32.0	9	33.3	9	32.1	36	34.6
Further education	9	37.5	8	32.0	7	25.9	9	32.1	33	31.7
University degree	5	20.8	9	36.0	11	40.7	10	35.7	35	33.7
Marital status										
Yes	17	70.8	21	84.0	21	77.8	23	82.1	82	78.8
No	7	29.2	4	16.0	6	22.2	5	17.9	22	21.2
PSA level ng/ml, median (IQR)	8.3	(5.8, 14.6)	8.4	(5.8, 12.4)	6.6	(5.2, 8.3)	8.1	(6.5, 12.5)	7.8	(5.8, 12.7)
Treatment group										
Active surveillance	11	45.8	12	48.0	13	48.1	12	42.9	48	46.2
Surgery	11	45.8	10	40.0	12	44.4	13	46.4	46	44.2
Radiotherapy	2	8.3	3	12.0	2	7.4	3	10.7	10	9.6
3 most common comorbidities										
Cholesterol	2	22.2	4	36.4	7	46.7	7	53.8	20	41.7
Asthma	4	44.4	5	45.5	4	26.7	1	7.7	14	29.2
Nervous trouble/depression	3	33.3	2	18.2	4	26.7	5	38.5	14	29.2
Taking medication										
Yes	19	79.2	18	72.0	20	74.1	23	82.1	80	76.9
No	3	12.5	7	28.0	7	25.9	4	14.3	21	20.2
Missing	2	8.3	0	0.0	0	0.0	1	3.6	3	2.9
Family history of prostate cancer										
Yes	0	0.0	4	16.0	5	18.5	5	17.9	14	13.5
No	17	70.8	14	56.0	19	70.4	17	60.7	67	64.4
Don't know/not answered	7	29.2	7	28.0	3	11.1	6	21.4	23	22.1
Height cm, mean (SD)	177.4	(6.0)	173.9	(17.1)	177.7	(6.6)	174.0	(14.0)	175.7	(11.8)
Weight kg, mean (SD)	86.1	(12.7)	86.6	(12.5)	87.1	(15.6)	85.6	(15.2)	86.3	(14.0)
BMI, mean (SD)	27.4	(3.9)	27.5	(3.4)	27.6	(4.8)	29.5	(9.3)	28.1	(6.0)
Smoking status										
Ever smoked	11	45.8	16	64.0	13	48.1	8	28.6	48	46.2
Never smoked	13	54.2	9	36.0	13	48.1	20	71.4	55	52.9
Missing	0	0.0	0	0.0	1	3.7	0	0.0	1	1.0
Alcohol units per week ^b^										
0–14 units per week	7	29.2	5	20.0	9	33.3	7	25.0	28	26.9
14 or more units per week	5	20.8	7	28.0	6	22.2	5	17.9	23	22.1
Missing	12	50.0	13	52.0	12	44.4	16	57.1	53	51.0

^a^Data includes men who were randomised to the interventions and consented at their baseline appointments (*n* = 104)

^b^One unit = 10 ml of pure alcohol

### Primary outcome—adherence

#### Physical activity

Forty-nine men were randomised to the physical activity intervention (± metformin) and of these 23 (46.9%) were adherent (95% CI 32.5 to 62.0) (Table 2). Adherent men also had a daily mean of 59.2 min (SD = 42.8, 95% CI 40.7 to 77.7) of brisk walking and 10,454.9 steps (SD = 4905.9, 95% CI: 8333.4 to 12,576.4). Non-adherent men had a daily mean of 22.6 min (*n* = 13, SD = 12.3, 95% CI 15.2 to 30.0) brisk walking on fewer than 5 days and achieved 7333.2 steps (SD = 1886.9, 95% CI 6193.0 to 8473.4). There were also 13 men with unknown duration of brisk walking and 2 provided step count data only (mean = 13,100.2, SD = 4525.8). In 23 men (43.4%) who provided their duration of brisk walking (*n* = 36) adherence was greater at 63.9% (95% CI 46.2 to 79.2).

**Table 2:** Physical activity adherence at 6 months post-randomisation

	Participants (percentage adherent)				Minutes per day (self-report)				Steps per day (pedometer)			
	*N*	%	95% LCI	95% UCI	Mean	SD	95% LCI	95% UCI	Mean	SD	95% LCI	95% UCI
Adherence groups												
Adherent^a^	23	46.9	32.5	62.0	59.2	42.8	40.7	77.7	10,454.9	4905.9	8333.4	12,576.4
Non-adherent^b^	13	26.5	14.9	41.1	22.6	12.3	15.2	30.0	7333.2	1886.9	6193.0	8473.4
Missing data (duration)^c^	13	26.5							13,100.2^d^	4525.8^d^		
Total	49	100.0			46.0	39.0	35.3	56.7	9624.9	4401.2		

^a^Brisk walked for ≥ 30 min in total on ≥ 5 days a week

^b^Brisk walked for ≤ 30 min in total on < 5 days a week

^c^Missing duration (in minutes) data

^d^Step counts of 2 participants with missing duration data

Fourteen men (14/49, 29.0%) in the brisk walking interventions reported their step counts and duration of brisk walking outside the intervention period at a mean follow-up of 10 months (mean days = 273.14, SD = 47.90). Nine of these men (64.3%) reported their brisk walking as ‘same as usual’ or ‘more’ during their delayed follow-up clinic appointments while two (14.3%) reported being less adherent.

#### Metformin

Fifty-one men were randomised to the metformin intervention (± physical activity) and of these 24 were adherent (47.1%, 95% CI 32.9 to 61.5). In the secondary outcome of self-reported adherence, 23 were adherent (45.1, 95% CI 31.1 to 59.7) (Table 3). Metformin adherence of men who were given tablets at 3 months post-randomisation (*n* = 38) before COVID-19 restrictions prevented clinics and resupply of tablets was 63.2% (95% CI 46.0 to 78.2) and 61.0% (95% CI 43.4 to 76.0) based on tablet counts and self-report adherence respectively.

**Table 3:** Metformin adherence at 6 months post-randomisation

	*n*	%	95% LCI	95% UCI
Tablet count adherence				
Tablets taken ≥ 60% between clinic visits^a^	24	47.1	32.9	61.5
Tablets counts not reported^b^	14	27.5	15.9	41.7
Tablets not administered (COVID-19)	9	17.7	8.4	30.9
Missing data^c^	4	7.8	2.2	18.9
Total	51	100.0		
Self-report adherence				
Adherent (≥ 5 days a week)	23	45.1	31.1	59.7
Non-adherent (0–4 days a week)	2	3.9	0.4	13.5
Tablets not administered (COVID-19)	9	17.7	8.4	30.9
Missing data	17	33.3	20.8	47.92
Total	51	100.0		

^a^3- and 6 months post-randomisation

^b^Tablets given but counts not reported at either 3- or 6 months post-randomisation

^c^Tablets given not reported

*LCI *Lower Confidence Interval, *UCI* Upper Confidence Interval

### Adverse events

Seven adverse (6.7%) events were reported: 4 in the physical activity and metformin intervention (gastric symptoms (*n* = 2), worsening of erectile dysfunction (*n* = 1), and heel pain (*n* = 1)); 2 in the metformin intervention (diarrhoea and nausea (*n* = 1), muscle pain in legs (*n* = 1)); and 1 in the control group (injured wrist unrelated to intervention). One serious adverse event was reported in the physical activity intervention and was categorised as unlikely related to the intervention.

### Patient-reported physical activity at 6 months

Physical activity levels were somewhat higher in the combined physical activity/metformin intervention (54.2% classed as active, 95% CI 32.8 to 74.4) and physical activity intervention (56.0%, 95% CI 34.9 to 75.6) compared to the metformin only (33.3%, 95% CI 16.5 to 54.0) and control groups (39.3%, 95% CI 22.0 to 59.4). Only two men in the metformin only group were categorised as insufficiently active. Physical activity levels could not be assessed for approximately 40% of men across all groups due to missing data (Table 4).

**Table 4:** Secondary outcomes at 6 months post-randomisation

	Intervention			
	Brisk walking and metformin	Brisk walking	Metformin	Control
	(*n* = 24)	(*n* = 25)	(*n* = 27)	(*n* = 28)
GSLTPAQ, *n* (%)				
Active	13 (54.2%)	14 (56.0%)	9 (33.3%)	11 (39.3%)
Moderately active	2 (8.3%)	1 (4.0%)	4 (14.8%)	5 (17.9%)
Insufficiently active	0 (0.0%)	0 (0.0%)	2 (7.4%)	0 (0.0%)
Missing	9 (37.5%)	10 (40.0%)	12 (44.4%)	12 (42.9%)
GSLTPAQ-frequency of activity (sweat), *n* (%)				
Often	8 (33.3%)	5 (20.0%)	9 (33.3%)	3 (10.7%)
Sometimes	11 (45.8%)	17 (68.0%)	3 (11.1%)	15 (53.6%)
Never/rarely	1 (4.2%)	0 (0.0%)	9 (33.3%)	4 (14.3%)
Missing	4 (16.7%)	3 (12.0%)	6 (22.2%)	6 (21.4%)
Adapted stages of change questionnaire, *n* (%)				
Pre-contemplation	1 (4.2%)	2 (8.0%)	5 (18.5%)	4 (14.3%)
Preparation	0 (0.0%)	0 (0.0%)	0 (0.0%)	1 (3.6%)
Action	4 (16.7%)	4 (16.0%)	3 (11.1%)	6 (21.4%)
Maintenance	12 (50.0%)	14 (56.0%)	5 (18.5%)	8 (28.6%)
Missing	7 (29.2%)	5 (20.0%)	14 (51.9%)	9 (32.1%)
Adapted theory of planned behaviour questionnaire, mean (SD) ^a^				
Attitude (good)	6.6 (0.6)	6.3 (1.2)	5.8 (1.6)	6.2 (1.3)
Attitude (10 K steps, good)	6.7 (0.6)	6.3 (1.2)	6.0 (1.5)	6.2 (1.5)
Attitude (pleasant)	5.8 (1.1)	5.2 (2.1)	4.9 (2.0)	4.5 (2.1)
Attitude (10 K steps, pleasant)	5.4 (1.6)	4.7 (2.0)	5.1 (2.0)	4.3 (2.4)
Perceived norm (injunctive)	6.5 (1.1)	6.0 (1.9)	6.1 (2.0)	6.5 (1.3)
Perceived norm (10 K steps, injunctive)	6.1 (1.7)	6.2 (1.6)	6.1 (1.7)	6.0 (1.7)
Perceived norm (descriptive)	5.6 (1.3)	4.2 (1.8)	5.3 (1.6)	4.4 (1.8)
Perceived norm (10 K steps, descriptive)	5.5 (1.5)	4.4 (2.0)	5.2 (1.6)	4.5 (1.9)
Perceived behavioural control (capacity)	6.4 (1.1)	5.6 (1.9)	5.3 (2.2)	5.2 (2.2)
Perceived behavioural control (10 K step, capacity),	5.2 (2.0)	5.0 (2.1)	6.0 (1.8)	4.4 (2.5)
Perceived behavioural control (10 K steps, autonomy)	6.2 (1.8)	6.4 (1.4)	6.9 (0.5)	6.6 (1.1)
Perceived behavioural control (autonomy)	6.8 (0.4)	6.4 (1.4)	6.8 (0.7)	6.7 (0.9)
Intention (PA)	6.0 (1.5)	5.0 (1.9)	3.7 (2.1)	4.0 (2.0)
Intention (10 K steps)	5.1 (2.1)	4.8 (2.0)	4.7 (1.9)	4.0 (2.5)
Past behaviour (PA)	5.3 (2.5)	5.5 (1.9)	4.0 (2.6)	4.2 (2.3)
Past behaviour (10 K steps)	5.3 (2.3)	4.9 (2.0)	5.0 (2.3)	4.6 (2.4)
Profile of mood states, median (IQR) ^b^				
Tension	5.0 (2.0, 6.0)	2.0 (2.0, 5.0)	2.0 (1.0, 3.0)	3.0 (2.0, 7.0)
Depression	4.0 (1.0, 6.0)	1.0 (0.0, 4.0)	1.0 (0.0, 2.5)	2.5 (0.0, 8.0)
Anger	2.5 (0.0, 5.0)	1.5 (0.0, 4.0)	1.0 (0.0, 2.0)	2.0 (0.0, 6.0)
Fatigue	4.0 (0.0, 8.0)	3.0 (0.0, 6.0)	2.0 (1.0, 7.0)	2.0 (0.5, 7.0)
Confusion	4.0 (2.0, 7.0)	3.0 (2.0, 4.0)	4.0 (2.0, 5.0)	4.0 (2.0, 7.0)
Vigour	18.0 (13.0, 22.0)	20.0 (16.0, 23.0)	18.0 (15.5, 22.0)	18.0 (13.0, 20.0)
Total mood disturbance	32.5 (21.0, 46.0)	29.0 (19.0, 36.0)	26.0 (0.0, 36.0)	26.5 (4.0, 37.0)
Benefit finding^a^	43.8 (18.3)	52.6 (15.2)	47.2 (13.7)	44.1 (17.8)
ICSmaleSF, median (IQR) ^b^				
Voiding	6.0 (4.0, 7.0)	4.0 (1.0, 6.0)	4.0 (2.0, 6.0)	4.5 (2.0, 7.0)
Incontinence	2.0 (2.0, 5.0)	2.0 (1.0, 5.5)	3.0 (3.0, 6.0)	3.5 (2.0, 5.0)
EPIC-26, median (IQR) ^a^				
Urinary incontinence	93.8 (60.5, 100.0)	85.5 (58.5, 100.0)	82.4 (60.5, 100.0)	89.6 (58.5, 100.0)
Urinary irritative/obstructive	84.4 (75.0, 93.8)	87.5 (81.2, 100.0)	93.8 (75.0, 93.8)	87.5 (81.2, 93.8)
Bowel	95.8 (85.4, 100.0)	95.8 (87.5, 100.0)	100.0 (95.8, 100.0)	100.0 (91.7, 100.0)
Sexual	16.7 (8.3, 57.0)	16.7 (5.5, 87.5)	44.5 (16.7, 66.7)	20.0 (8.3, 62.5)
Hormonal	95.0 (90.0, 100.0)	95.0 (90.0, 100.0)	95.0 (90.0, 100.0)	95.0 (85.0, 100.0)
FACT, mean (SD) ^a^				
Physical well-being	25.1 (3.3)	25.1 (3.3)	26.0 (2.4)	24.3 (3.6)
Social well-being	17.8 (5.4)	19.7 (6.6)	20.4 (4.1)	18.7 (5.9)
Emotional well-being	20.8 (2.0)	20.3 (3.8)	20.8 (3.1)	19.6 (3.8)
Functional well-being	23.0 (3.6)	23.2 (5.0)	23.5 (3.2)	20.5 (5.7)
FACT-Prostate, mean (SD) ^a^	122.2 (13.2)	124.4 (21.3)	129.5 (14.4)	117.5 (18.7)
FACIT-Fatigue, mean (SD) ^a^	131.5 (16.3)	132.9 (22.1)	135.9 (15.8)	125.4 (21.9)
EQ-5D-5L, mean (SD) ^a^				
EQ-5D index value	0.9 (0.1)	0.9 (0.1)	0.9 (0.1)	0.9 (0.2)
EQ VAS	81.5 (9.5)	81.6 (16.2)	81.6 (10.0)	82.0 (11.7)
Weight (kg), mean (SD)	85.5 (14.7)	84.3 (12.3)	85.5 (15.5)	86.6 (17.6)
BMI, mean (SD)	27.3 (4.9)	27.2 (2.9)	27.3 (4.3)	30.0 (6.1)
Smoking status, *n* (%)				
Ever smoked	8 (33.3%)	15 (60.0%)	10 (37.0%)	6 (21.4%)
Never smoked	12 (50.0%)	7 (28.0%)	11 (40.7%)	17 (60.7%)
Missing	4 (16.7%)	3 (12.0%)	6 (22.2%)	5 (17.9%)
Alcohol units per week, *n* (%)				
< 14 units	5 (20.8%)	7 (28.0%)	7 (25.9%)	12 (42.9%)
> = 14 units	5 (20.8%)	4 (16.0%)	4 (14.8%)	3 (10.7%)
Missing	14 (58.3%)	14 (56.0%)	16 (59.3%)	13 (46.4%)

*Abbreviations*: *N* number, *SD* standard deviation, *IQR* interquartile range, *BW* brisk walking, *PA* physical activity, *kg* kilograms, *GLTPAQ* Godin-Shephard Leisure-time physical activity questionnaire, *ICSmaleSF* International Continence Society male short form, *EPIC-26* Expanded Prostate Cancer Index Composite–26, *FACT* Functional Assessment of Cancer Therapy, *FACIT* Functional Assessment of Chronic Illness Therapy, *EQ VAS* EQ visual analogue scale

^a^Higher scores represent better outcomes

^b^Higher scores represent worse outcomes

^c^One unit = 10 ml of pure alcohol

#### Feasibility of activity monitors and effect on step counts

Two thirds of men (*n* = 25/39, 64.1%) in the physical activity interventions who were offered the wrist-worn activity monitor consented to wear it for 6 months. However, all men were given a pedometer and step counts were comparable between men with and without activity monitors (*n* = 14, mean = 9635.5, SD = 4102.8, 95% CI 2265.1 to 16,300.4; *n* = 21, mean = 9617.9, SD = 4689.1, 95% CI 4176.6 to 25,808.9, respectively) at 6 months suggesting that they did not act as motivational tools for exercise.

### Patient reported secondary outcomes

Completion rates across all randomised groups were high at 6 months across a range of measures: 70.9% and 98.9% (GSLTPAQ and its frequency of activity item, respectively). All other measures were at least 80.0% and some were at least 95.0% (Benefit Finding Scale, ICSmale-Short form, EPIC-26, FACIT-Fatigue and FACT-prostate subscales and EQ-5D-5L. Supplementary Table S2 (Additional file 2) presents baseline and later follow-ups.

Patient reported outcomes at 6 months post-randomisation were similar between randomised groups (Table 4). However, 50.0% (95% CI 29.1 to 70.9) in the combined physical activity/metformin intervention and 56.0% (95% CI 34.9 to 75.6) reported maintaining the brisk walking at 6 months compared to the metformin intervention (18.5%, 95% CI 6.3 to 38.1) and control group (28.6%, 95% CI 13.2 to 48.7). All other outcomes were comparable between randomised groups at all remaining follow-ups (Additional file 2).

For men who underwent surgery (*n* = 46), those in the brisk walking intervention had higher urinary voiding symptoms (median = 7.0, IQR = 1.0, 10.0) compared to the combined brisk walking/metformin intervention (median = 3.0 (IQR = 1.0, 5.0), metformin intervention (median = 1.0, IQR = 1.0, 3.5) and control group (median = 4.0, IQR = 1.0, 6.0) at 7 weeks post-randomisation. Men in the brisk walking (median = 9.0, IQR = 1.0, 11.0) and metformin intervention (median = 9.0, IQR = 4.0, 12.5) had higher urinary continence symptoms compared to the combined brisk walking/metformin intervention (median = 7.5, IQR = 5.0, 9.5) and control group (median = 6.0, IQR = 4.0, 7.0). Suitable comparisons in fatigue and quality of life scores could not be made for men in the radiotherapy treatment group as a very small number of men completed questionnaires at 4.5 months post-randomisation (*n* = 5) (Supplementary Table S1, Additional file 2).

#### Patient characteristics at 6 months

Smoking status, alcohol intake, weight and BMI were comparable across all intervention groups at 6 months post-randomisation (Table 4). Supplementary Tables S2–S3 present 3 and 12 months post-randomisation data.

## Discussion

This study established the potential of randomising men with localised or locally advanced prostate cancer to physical activity and/or metformin interventions. Our randomisation rate of eligible men was 37%, which exceeded the progression criterion to a main trial of ≥ 20%. This rate is comparable to our previous factorial trial (35% [31]), which included physical activity and nutritional interventions but not a prescribed medication.

Adherence to the physical activity interventions was below our predefined criterion of 50% based on frequency combined with duration of brisk walking as measured by pedometers. This finding contrasts with another feasibility randomised controlled trial, which showed that 50% of men with prostate cancer on Androgen Deprivation Therapy (ADT) adhered to 150 min of home-based physical activity (accelerometer measured) a week over 6 months follow-up [32]. and for other types of treatment. A study randomising men with prostate cancer to moderate-vigorous home-based resistance exercise following radical prostatectomy reported an adherence rate of 90% (defined as number of sessions completed) at 6 months follow-up [10]. A similar percentage of men treated with radical prostatectomy or under active surveillance were adherent to home-based endurance physical activity at a 2-year follow-up [33]. Notably in these studies was the level of contact with the research teams (i.e. weekly phone calls, monthly attendance to research visits). In addition, a systematic review reported an average adherence rate of 80% in men with prostate cancer attending mostly supervised exercise sessions [34]. Regular peer-support is likely to be an important to adherence to physical activity interventions in men with prostate cancer [35]. We were unable to prompt approximately 20% of men around 3 and 5 months of the intervention period due to COVID-19 restrictions, which may have impacted on intervention adherence. Most men in the physical activity interventions consented to wear activity monitors although they did not increase men’s step counts as a motivational tool. This finding is supported by qualitative studies conducted in older adults which have shown that step counts are of interest to participants with gauging their level of physical activity, but do not incentivise them to increase their physical activity [36]. A systematic review of randomised controlled trials studies involving cancer patients found that wearable activity monitors had a moderate effect on daily steps (Standardised mean difference = 0.54, 95% CI 0.30 to 0.78) [37]. These findings suggest future studies should consider other measures of physical activity, such as minutes of exercise, when setting goals to increase motivation in prostate cancer patients.

Adherence to metformin (45%) did not meet our predefined criterion of ≥ 60% at 6 months follow-up, which is significantly lower compared to other prostate cancer studies using supplements as preventative therapies [38]. However, tablet counts for 26% of men who were given metformin at 3 months follow-up were missing and the reasons are unknown. However, it does suggest other methods of capturing medication adherence at follow-ups are needed. Digital devices, which can capture real-time medication adherence, could offer a way of reducing missing data collected at follow-up [39]. Nevertheless, few adverse events reported in our study demonstrate that metformin was well-tolerated in men with non-metastatic prostate cancer.

The strengths of our study include a factorial RCT design allowing two interventions to be assessed concurrently, good recruitment of men from three main prostate cancer treatments, multiple outcomes measures (e.g. self-report questionnaires, blood samples, qualitative interviews), and participant retention (88%). However, the study has several limitations. We are unable to make comparisons between eligible non-participants to study participants as demographic data was collected following consent to participate in the study. Such comparisons would have determined whether study participants differed significantly from non-participant in terms of healthy lifestyle factors, which may have impacted on their willingness to be recruit into the study. Recruitment stopped early due to the COVID-19 pandemic which impaired assessing adherence, particular metformin, at 6 months follow-up. Approximately, 30% of men did not report the duration of their brisk walking, which meant that adherence could not be calculated for these men. We were unable to assess physical activity levels for a significant proportion of men (~ 40%) due to missing items on this outcome measure. A systematic review [40] showed that the validity of the GSLTPAQ scoring of physical activity had not been assessed in cancer populations and 80% of cancer studies had modified the scoring method which may have improved the performance of the questionnaire. Further research on assessing the validity of the GSLTPAQ may be required before using in prostate cancer populations. Completion rates were high among all other questionnaires, which indicates their feasibility in this population. Also, a percentage of the data was collected outside the 6 months intervention period and it is possible men were not adhering to the intervention. We did not plan to use the wrist-worn monitor data for assessing physical activity which could have supplemented or replaced the missing data from self-reported data. Future studies would benefit from the use of application programming interfaces (APIs) that enables study teams to remotely access participants’ real-time physical activity data. APIs remove the need for participants to manually record their physical activity data, reduce the risk of missing data, and enable the monitoring of intervention compliance. APIs have been effectively used in trials which have increased physical activity levels in cancer patients [41, 42]. Most men were of white ethnicity (91.3%) so these findings may not represent those from men from other ethnic groups. We also recruited few men undergoing radiotherapy, which may be due to men with localised or locally advanced disease more frequently selecting radical prostatectomy or active surveillance.

## Conclusions

To conclude, our study was successful at randomising men with prostate cancer to a physical activity and metformin intervention for 6 months. However, further consideration on the measures of adherence and motivational support received by men during these interventions is required before progressing to a larger scale trial with clinical endpoints.

## Supplementary Information

Additional file 1. CONSORT checklist.doc.

Additional file 2: Supplementary Table S1. Secondary outcome measures completed 7-weeks and 4.5 months post-randomisation by men treated with radical prostatectomy and radiotherapy, respectively. Supplementary Table S2. Questionnaire completion rates at each time point. Supplementary Table S3. Baseline secondary outcomes. Supplementary Table S4. Secondary outcomes at 3 months post-randomisation. Supplementary Table S5. Secondary outcomes at 12 months post-randomisation.

## Data Availability

The datasets used and/or analysed during the current study are available from the corresponding author on reasonable request.

## Abbreviations

ADT: Androgen Deprivation Therapy
BMI: Body Mass Index
eGFR: Estimated glomerular filtration rate
EPIC-26: Expanded Prostate cancer Index Composite-26
FACIT–Fatigue: Functional Assessment of Chronic Illness Therapy-Fatigue
FACT-P: Functional Assessment of Cancer Therapy–Prostate
GSLTPAQ: Godin-Shepard Leisure-time Physical Activity Questionnaire
ICSmale-SF: International Continence Society male–Short Form
ITT: Intention-to-treat
NHS: National Health Service
POMS: Profile of Mood States–Short Form
Pre-EMpT: Prostate cancer–Exercise and Metformin Trial
PSA: Prostate specific antigen
RCT: Randomised controlled trial
REC: Research Ethics Committee
REDCap: Research Electronic Data Capture
