# Supplementary material for: The Prostate cancer–Exercise and Metformin randomised controlled feasibility Trial (Pre-EMpT) in men following active surveillance, radical prostatectomy and radiotherapy
Source: Pilot Feasibility Stud. 2025 May 24;11:71. doi: 10.1186/s40814-025-01654-0 (PMC12103774; doi:10.1186/s40814-025-01654-0)
Supplement: Supplementary file 2 — Additional file 2: Supplementary Table S1. Secondary outcome measures completed 7-weeks and 4.5 months post-randomisation by men treated with radical prostatectomy and radiotherapy, respectively. Supplementary Table S2. Questionnaire completion rates at each time point. Supplementary Table S3. Baseline secondary outcomes. Supplementary Table S4. Secondary outcomes at 3 months post-randomisation. Supplementary Table S5. Secondary outcomes at 12 months post-randomisation. [file 40814_2025_1654_MOESM2_ESM.docx]

**Additional file 2. Supplementary tables**

| **Supplementary table 1. Secondary outcome measures completed 7-weeks and 4.5 months post-randomisation by men treated with radical prostatectomy and radiotherapy, respectively** | | | | |
| --- | --- | --- | --- | --- |
|  | Intervention | | | |
|  | Brisk Walking and Metformin | Brisk Walking | Metformin | Control |
|  | (n = 11) | (n = 10) | (n = 12) | (n = 13) |
| *Radical prostatectomy* |  |  |  |  |
| ICSmaleSF, median (IQR) ^b^ |  |  |  |  |
| ICS void | 3.0 (1.0, 5.0) | 7.0 (1.0, 10.0) | 1.0 (1.0, 3.5) | 4.0 (1.0, 6.0) |
| ICS continence | 7.5 (5.0, 9.5) | 9.0 (1.0, 11.0) | 9.0 (4.0, 12.5) | 6.0 (4.0, 7.0) |
|  | Brisk Walking and Metformin | Brisk Walking | Metformin | Control |
|  | (n = 2) | (n = 3)^c^ | (n = 2)^d^ | (n = 3) |
| *Radiotherapy* |  |  |  |  |
| FACIT-F, mean (SD) ^a^ | 36.5 (12.0) | -- | 50.0 (.) | 36.0 (1.4) |
| EQ-5D-5L, median (IQR) ^a^ |  |  |  |  |
| EQ-5D index value | 0.8 (0.7, 0.9) | -- | 0.9 (0.9, 0.9) | 0.9 (0.9, 0.9) |
| EQ VAS | 65.0 (50.0, 80.0) | -- | 80.0 (80.0, 80.0) | 60.0 (50.0, 70.0) |
| Abbreviations: N, number; IQR, interquartile range; SD, standard deviation; ICSmale-SF, International Continence Society male - Short form FACIT-F, Functional Assessment of Chronic Illness Therapy - Fatigue; EQ VAS, EQ visual analogue scale.  ^a^ Higher scores represent better outcomes.  ^b^ Higher scores represent worse outcomes.  ^c^ Not data collected on participants in the brisk walking intervention.  ^d^ No SD as data collected on one participant only. | | | | |

| **Supplementary table 2. Questionnaire completion rates at each time point** | | | | |
| --- | --- | --- | --- | --- |
|  | Baseline | 3 months | 6 months | 12 months |
|  | (n = 104) | (n = 74) | (n = 86) | (n = 70) |
| GSLTPAQ | 63 (60.6%) | 51 (68.9%) | 61 (70.9%) | 48 (68.6%) |
| GSLTPAQ - Frequency of activity (sweat) | 103 (99.0%) | 74 (100.0%) | 85 (98.8%) | 69 (98.6%) |
| Adapted stages of change questionnaire | 77 (74.0%) | 55 (74.3%) | 69 (80.2%) | 53 (75.7%) |
| Adapted theory of planned behaviour questionnaire | |  |  |  |
| Attitude (BW, good) | 102 (98.1%) | 73 (98.6%) | 81 (94.2%) | 66 (94.3%) |
| Attitude (10K steps, good) | 102 (98.1%) | 72 (97.3%) | 81 (94.2%) | 68 (97.1%) |
| Attitude (BW, pleasant) | 86 (82.7%) | 65 (87.8%) | 71 (82.6%) | 65 (92.9%) |
| Attitude (10K steps, pleasant) | 85 (81.7%) | 65 (87.8%) | 72 (83.7%) | 64 (91.4%) |
| Perceived norm (BW, injunctive) | 101 (97.1%) | 72 (97.3%) | 82 (95.3%) | 69 (98.6%) |
| Perceived norm (10K steps, injunctive) | 101 (97.1%) | 72 (97.3%) | 84 (97.7%) | 69 (98.6%) |
| Perceived norm (BW, descriptive) | 100 (96.2%) | 72 (97.3%) | 82 (95.3%) | 69 (98.6%) |
| Perceived norm (10K steps, descriptive) | 99 (95.2%) | 74 (100.0%) | 83 (96.5%) | 69 (98.6%) |
| Perceived behaviour control (BW, capacity) | 102 (98.1%) | 73 (98.6%) | 84 (97.7%) | 69 (98.6%) |
| Perceived behavioural control (10K steps, capacity) | 102 (98.1%) | 72 (97.3%) | 85 (98.8%) | 68 (97.1%) |
| Perceived behaviour control (BW, autonomy) | 102 (98.1%) | 73 (98.6%) | 86 (100.0%) | 69 (98.6%) |
| Perceived behavioural control (10K steps, autonomy) | 102 (98.1%) | 74 (100.0%) | 86 (100.0%) | 69 (98.6%) |
| Intention (10K steps) | 100 (96.2%) | 71 (95.9%) | 84 (97.7%) | 70 (100.0%) |
| Intention (BW) | 98 (94.2%) | 72 (97.3%) | 83 (96.5%) | 69 (98.6%) |
| Past behaviour (10K steps) | 100 (96.2%) | 73 (98.6%) | 85 (98.8%) | 70 (100.0%) |
| Past behaviour (BW) | 101 (97.1%) | 72 (97.3%) | 85 (98.8%) | 70 (100.0%) |
| Profile of mood states |  |  |  |  |
| Tension | 98 (94.2%) | 67 (90.5%) | 83 (96.5%) | 66 (94.3%) |
| Depression | 88 (84.6%) | 60 (81.1%) | 78 (90.7%) | 64 (91.4%) |
| Anger | 93 (89.4%) | 62 (83.8%) | 77 (89.5%) | 66 (94.3%) |
| Fatigue | 96 (92.3%) | 65 (87.8%) | 76 (88.4%) | 65 (92.9%) |
| Confusion | 94 (90.4%) | 66 (89.2%) | 80 (93.0%) | 68 (97.1%) |
| Vigour | 94 (90.4%) | 65 (87.8%) | 76 (88.4%) | 68 (97.1%) |
| Total mood disturbance |  |  |  |  |
| Benefit finding | 100 (96.2%) | 71 (95.9%) | 84 (97.7%) | 68 (97.1%) |
| ICSmaleSF |  |  |  |  |
| ICS void | 104 (100.0%) | 74 (100.0%) | 84 (97.7%) | 70 (100.0%) |
| ICS continence | 104 (100.0%) | 74 (100.0%) | 84 (97.7%) | 70 (100.0%) |
| EPIC-26 |  |  |  |  |
| Urinary | 101 (97.1%) | 71 (95.9%) | 82 (95.3%) | 69 (98.6%) |
| Irritative | 98 (94.2%) | 70 (94.6%) | 82 (95.3%) | 66 (94.3%) |
| Bowel | 102 (98.1%) | 73 (98.6%) | 86 (100.0%) | 69 (98.6%) |
| Sexual | 102 (98.1%) | 72 (97.3%) | 83 (96.5%) | 68 (97.1%) |
| Hormonal | 102 (98.1%) | 73 (98.6%) | 84 (97.7%) | 67 (95.7%) |
|  |  |  |  |  |
| FACIT-F | 101 (97.1%) | 73 (98.6%) | 85 (98.8%) | 67 (95.7%) |
| FACT-P | 101 (97.1%) | 72 (97.3%) | 85 (98.8%) | 68 (97.1%) |
| EQ-5D-5L |  |  |  |  |
| EQ-5D index value | 101 (97.1%) | 74 (100.0%) | 82 (95.3%) | 68 (97.1%) |
| EQ VAS | 104 (100.0%) | 74 (100.0%) | 85 (98.8%) | 69 (98.6%) |
| Abbreviations: N, number; SD, standard deviation; IQR, interquartile range; BW, brisk walking; PA, physical activity; GLTPAQ, Godin-Shephard Leisure-time physical activity questionnaire; ICSmaleSF, International Continence Society male short form; EPIC-26, Expanded Prostate Cancer Index Composite – 26; FACT, Functional Assessment of Cancer Therapy; FACIT, Functional Assessment of Chronic Illness Therapy; EQ VAS, EQ visual analogue scale. | | | | |

| **Supplementary table 3. Baseline secondary outcomes** | | | | |
| --- | --- | --- | --- | --- |
|  | Intervention | | | |
|  | Brisk Walking and Metformin | Brisk Walking | Metformin | Control |
|  | (n = 24) | (n = 25) | (n = 27) | (n = 28) |
| GSLTPAQ, n (%) |  |  |  |  |
| Active | 9 (37.5%) | 11 (44.0%) | 12 (44.4%) | 9 (32.1%) |
| Moderately active | 2 (8.3%) | 3 (12.0%) | 4 (14.8%) | 4 (14.3%) |
| Insufficiently active | 3 (12.5%) | 1 (4.0%) | 5 (18.5%) | 0 (0.0%) |
| Missing | 10 (41.7%) | 10 (40.0%) | 6 (22.2%) | 15 (53.6%) |
| GSLTPAQ - Frequency of activity (sweat), n (%) |  |  |  |  |
| Often | 7 (29.2%) | 8 (32.0%) | 8 (29.6%) | 3 (10.7%) |
| Sometimes | 16 (66.7%) | 10 (40.0%) | 13 (48.1%) | 19 (67.9%) |
| Never/rarely | 1 (4.2%) | 6 (24.0%) | 6 (22.2%) | 6 (21.4%) |
| Missing | 0 (0.0%) | 1 (4.0%) | 0 (0.0%) | 0 (0.0%) |
| Adapted stages of change questionnaire, n (%) |  |  |  |  |
| Pre-contemplation | 6 (25.0%) | 6 (24.0%) | 13 (48.1%) | 13 (46.4%) |
| Preparation | 2 (8.3%) | 0 (0.0%) | 3 (11.1%) | 0 (0.0%) |
| Action | 2 (8.3%) | 4 (16.0%) | 2 (7.4%) | 3 (10.7%) |
| Maintenance | 6 (25.0%) | 11 (44.0%) | 2 (7.4%) | 4 (14.3%) |
| Missing | 8 (33.3%) | 4 (16.0%) | 7 (25.9%) | 8 (28.6%) |
| Adapted theory of planned behaviour questionnaire, mean (SD) ^a^ |  |  |  |  |
| Attitude (good) | 6.2 (1.0) | 6.4 (0.8) | 6.4 (1.4) | 6.6 (0.7) |
| Attitude (10K steps, good) | 6.1 (1.3) | 6.6 (0.7) | 6.5 (1.2) | 6.7 (0.6) |
| Attitude (pleasant) | 5.1 (2.0) | 5.7 (1.5) | 5.7 (1.7) | 5.4 (1.8) |
| Attitude (10K steps, pleasant) | 5.0 (1.8) | 5.6 (1.7) | 5.8 (1.4) | 4.8 (2.1) |
| Perceived norm (injunctive) | 6.3 (1.3) | 6.4 (1.7) | 6.1 (1.6) | 5.6 (2.3) |
| Perceived norm (10K steps, injunctive) | 6.0 (1.6) | 6.6 (1.3) | 6.3 (1.4) | 5.6 (2.2) |
| Perceived norm (descriptive) | 4.6 (1.5) | 4.1 (2.1) | 4.4 (2.2) | 4.1 (2.0) |
| Perceived norm (10K steps, descriptive) | 5.0 (1.6) | 4.5 (2.0) | 4.8 (2.1) | 4.4 (1.7) |
| Perceived behavioural control (capacity) | 6.2 (1.6) | 6.4 (1.1) | 6.3 (1.0) | 5.5 (2.3) |
| Perceived behavioural control (10K steps, capacity) | 5.4 (2.2) | 6.2 (1.2) | 6.1 (1.4) | 5.5 (2.2) |
| Perceived behavioural control (autonomy) | 6.5 (0.7) | 6.5 (0.8) | 6.3 (1.2) | 6.8 (0.5) |
| Perceived behavioural control (10K steps, autonomy) | 6.4 (0.9) | 6.6 (0.8) | 6.5 (1.0) | 6.9 (0.5) |
| Intention (PA) | 5.8 (1.7) | 5.2 (2.1) | 5.5 (1.7) | 5.5 (2.2) |
| Intention (10K steps) | 5.3 (1.9) | 5.7 (1.6) | 5.1 (2.1) | 5.7 (2.0) |
| Past behaviour (PA) | 3.8 (2.4) | 5.1 (2.3) | 4.6 (2.5) | 4.0 (2.5) |
| Past behaviour (10K steps) | 4.0 (2.2) | 5.4 (2.1) | 4.5 (2.4) | 4.7 (2.3) |
| Profile of mood states, median (IQR) ^b^ |  |  |  |  |
| Tension | 3.0 (1.0, 6.0) | 2.0 (1.0, 4.0) | 3.0 (1.0, 6.0) | 6.0 (2.0, 7.0) |
| Depression | 0.0 (0.0, 3.0) | 1.0 (0.0, 3.0) | 1.0 (0.0, 2.0) | 2.0 (1.0, 4.0) |
| Anger | 1.0 (0.0, 4.0) | 1.0 (0.0, 3.0) | 1.0 (0.0, 5.0) | 2.0 (0.0, 5.0) |
| Fatigue | 2.0 (0.5, 5.5) | 1.0 (0.0, 3.0) | 2.0 (0.0, 3.0) | 2.0 (1.0, 5.0) |
| Confusion | 3.0 (2.0, 5.5) | 4.0 (2.0, 6.0) | 3.0 (1.0, 6.0) | 4.0 (3.0, 5.0) |
| Vigour | 20.5 (16.0, 23.0) | 20.0 (16.0, 23.0) | 20.0 (15.0, 23.5) | 19.0 (17.0, 21.5) |
| Total mood disturbance | 31.0 (27.0, 36.0) | 29.0 (24.0, 36.0) | 30.0 (25.0, 38.0) | 33.5 (28.5, 40.5) |
| Benefit finding, mean (SD) ^a^ | 45.1 (17.2) | 50.6 (15.6) | 43.9 (15.2) | 43.4 (16.9) |
| ICSmaleSF, median (IQR) ^b^ |  |  |  |  |
| Voiding | 7.0 (4.5, 9.5) | 6.0 (3.0, 9.0) | 4.0 (2.0, 9.0) | 4.5 (3.0, 8.0) |
| Incontinence | 2.0 (1.0, 3.0) | 1.0 (0.0, 4.0) | 2.0 (1.0, 5.0) | 1.0 (1.0, 3.0) |
| EPIC-26, median (IQR) ^a^ |  |  |  |  |
| Urinary Incontinence | 100.0 (82.4, 100.0) | 100.0 (73.0, 100.0) | 91.8 (66.8, 100.0) | 100.0 (87.5, 100.0) |
| Urinary Irritative / Obstructive | 87.5 (68.8, 93.8) | 87.5 (68.8, 100.0) | 87.5 (75.0, 93.8) | 93.8 (81.2, 93.8) |
| Bowel | 95.8 (85.4, 100.0) | 100.0 (89.6, 100.0) | 100.0 (87.5, 100.0) | 100.0 (91.7, 100.0) |
| Sexual | 51.4 (31.2, 77.1) | 69.4 (44.5, 86.2) | 70.8 (34.7, 83.3) | 44.5 (36.2, 70.8) |
| Hormonal | 100.0 (87.5, 100.0) | 95.0 (85.0, 100.0) | 97.5 (90.0, 100.0) | 100.0 (92.5, 100.0) |
| FACT, mean (SD) ^a^ |  |  |  |  |
| Physical well-being | 25.5 (2.5) | 25.6 (2.1) | 25.5 (2.3) | 25.0 (3.3) |
| Social well-being | 19.7 (5.4) | 20.6 (6.3) | 19.2 (6.2) | 19.9 (6.1) |
| Emotional well-being | 19.5 (2.7) | 20.0 (3.2) | 18.9 (4.3) | 19.3 (3.5) |
| Functional well-being | 23.4 (4.9) | 22.5 (4.9) | 23.7 (3.4) | 22.0 (4.4) |
| FACT-Prostate, mean (SD) ^a^ | 124.6 (14.4) | 125.6 (18.0) | 124.4 (17.7) | 124.4 (14.9) |
| FACIT-Fatigue, mean (SD) ^a^ | 134.1 (16.8) | 134.8 (18.4) | 132.0 (18.9) | 132.5 (15.2) |
| EQ-5D-5L, mean (SD) ^a^ |  |  |  |  |
| EQ-5D index value | 0.9 (0.1) | 0.9 (0.1) | 0.9 (0.1) | 0.9 (0.1) |
| EQ VAS | 80.2 (9.5) | 83.4 (14.2) | 80.9 (11.7) | 77.5 (12.5) |
| Abbreviations: N, number; SD, standard deviation; IQR, interquartile range; BW, brisk walking; PA, physical activity; kg, kilograms; ; GLTPAQ, Godin-Shephard Leisure-time physical activity questionnaire; ICSmaleSF, International Continence Society male short form; EPIC-26, Expanded Prostate Cancer Index Composite – 26; FACT, Functional Assessment of Cancer Therapy; FACIT, Functional Assessment of Chronic Illness Therapy; EQ VAS, EQ visual analogue scale.  ^a^ Higher scores represent better outcomes.  ^b^ Higher scores represent worse outcomes.  ^C^ One unit = 10ml of pure alcohol. | | | | |

| **Supplementary table 4. Secondary outcomes at 3 months post-randomisation** | | | | |
| --- | --- | --- | --- | --- |
|  | Intervention | | | |
|  | Brisk Walking and Metformin | Brisk Walking | Metformin | Control |
|  | (n = 24) | (n = 25) | (n = 27) | (n = 28) |
| GSLTPAQ, n (%) |  |  |  |  |
| Active | 11 (45.8%) | 8 (32.0%) | 8 (29.6%) | 9 (32.1%) |
| Moderately active | 0 (0.0%) | 1 (4.0%) | 3 (11.1%) | 2 (7.1%) |
| Insufficiently active | 0 (0.0%) | 2 (8.0%) | 5 (18.5%) | 2 (7.1%) |
| Missing | 13 (54.2%) | 14 (56.0%) | 11 (40.7%) | 15 (53.6%) |
| GSLTPAQ - Frequency of activity (sweat), n (%) |  |  |  |  |
| Often | 6 (25.0%) | 4 (16.0%) | 6 (22.2%) | 0 (0.0%) |
| Sometimes | 9 (37.5%) | 11 (44.0%) | 9 (33.3%) | 11 (39.3%) |
| Never/rarely | 2 (8.3%) | 2 (8.0%) | 7 (25.9%) | 7 (25.0%) |
| Missing | 7 (29.2%) | 8 (32.0%) | 5 (18.5%) | 10 (35.7%) |
| Adapted stages of change questionnaire, n (%) |  |  |  |  |
| Pre-contemplation | 1 (4.2%) | 3 (12.0%) | 5 (18.5%) | 5 (17.9%) |
| Preparation | 0 (0.0%) | 1 (4.0%) | 0 (0.0%) | 1 (3.6%) |
| Action | 9 (37.5%) | 9 (36.0%) | 6 (22.2%) | 4 (14.3%) |
| Maintenance | 5 (20.8%) | 2 (8.0%) | 1 (3.7%) | 3 (10.7%) |
| Missing | 9 (37.5%) | 10 (40.0%) | 15 (55.6%) | 15 (53.6%) |
| Adapted theory of planned behaviour questionnaire, mean (SD) ^a^ |  |  |  |  |
| Attitude (good) | 6.4 (0.9) | 6.1 (1.8) | 5.6 (1.6) | 6.1 (1.0) |
| Attitude (10K steps, good) | 6.4 (0.8) | 6.2 (1.5) | 6.0 (1.4) | 6.4 (1.2) |
| Attitude (pleasant) | 5.5 (1.6) | 5.7 (1.9) | 5.1 (1.6) | 4.7 (1.9) |
| Attitude (10K steps, pleasant) | 5.1 (2.0) | 5.6 (2.1) | 5.2 (1.5) | 4.5 (2.2) |
| Perceived norm (injunctive) | 6.2 (1.6) | 6.5 (1.2) | 5.9 (1.9) | 5.9 (1.7) |
| Perceived norm (10K steps, injunctive) | 5.4 (2.3) | 6.2 (1.7) | 5.9 (1.6) | 5.9 (1.8) |
| Perceived norm (descriptive) | 4.9 (2.1) | 4.2 (1.8) | 5.0 (1.8) | 3.8 (2.0) |
| Perceived norm (10K steps, descriptive) | 5.3 (2.0) | 4.5 (1.8) | 5.3 (1.5) | 4.3 (1.9) |
| Perceived behavioural control (capacity) | 6.0 (1.7) | 5.9 (1.5) | 5.0 (2.0) | 4.5 (1.8) |
| Perceived behavioural control (10K steps, capacity) | 5.2 (2.2) | 5.5 (1.9) | 5.2 (1.8) | 4.4 (2.3) |
| Perceived behavioural control (autonomy) | 6.6 (0.9) | 6.2 (1.6) | 6.4 (1.0) | 6.6 (0.8) |
| Perceived behavioural control (10K steps, autonomy) | 6.4 (1.1) | 6.1 (1.6) | 6.5 (0.8) | 6.7 (1.0) |
| Intention (PA) | 5.9 (1.8) | 5.6 (1.7) | 4.1 (2.0) | 3.9 (1.8) |
| Intention (10K steps) | 5.4 (2.1) | 5.4 (1.7) | 4.3 (1.8) | 4.1 (2.4) |
| Past behaviour (PA) | 4.5 (2.3) | 5.1 (2.0) | 3.9 (2.4) | 3.8 (2.5) |
| Past behaviour (10K steps) | 4.4 (2.0) | 5.2 (1.9) | 4.7 (2.3) | 4.7 (2.2) |
| Profile of mood states, median (IQR) ^b^ |  |  |  |  |
| Tension | 4.0 (1.0, 6.0) | 3.0 (1.0, 6.0) | 2.0 (1.0, 4.0) | 5.0 (3.0, 8.0) |
| Depression | 3.0 (1.0, 6.0) | 2.0 (1.0, 4.0) | 0.0 (0.0, 1.0) | 2.0 (0.0, 3.5) |
| Anger | 2.0 (1.0, 4.0) | 2.5 (0.0, 4.0) | 1.0 (0.0, 3.0) | 3.0 (0.0, 8.0) |
| Fatigue | 2.5 (0.0, 7.0) | 3.5 (1.5, 7.5) | 3.0 (0.0, 6.0) | 5.0 (1.0, 8.0) |
| Confusion | 3.5 (1.5, 5.5) | 5.0 (3.0, 6.0) | 2.5 (1.0, 5.0) | 4.0 (3.0, 5.0) |
| Vigour | 19.5 (13.5, 23.5) | 20.0 (15.0, 23.0) | 17.5 (14.5, 22.0) | 15.5 (11.0, 17.0) |
| Total mood disturbance | 28.5 (0.0, 37.0) | 28.0 (0.0, 36.0) | 27.0 (3.0, 32.0) | 17.0 (0.0, 38.0) |
| Benefit finding, mean (SD) ^a^ | 47.6 (18.0) | 50.9 (15.5) | 43.2 (13.6) | 45.4 (18.6) |
| ICSmaleSF, median (IQR) ^b^ |  |  |  |  |
| Voiding | 5.0 (4.0, 8.0) | 5.0 (3.0, 7.0) | 4.0 (2.0, 5.0) | 4.0 (2.0, 5.0) |
| Incontinence | 5.0 (1.0, 6.0) | 6.0 (3.0, 7.0) | 4.0 (2.0, 7.0) | 2.0 (2.0, 5.0) |
| EPIC-26, median (IQR) ^a^ |  |  |  |  |
| Urinary Incontinence | 77.1 (56.4, 91.8) | 60.5 (53.1, 79.2) | 76.1 (46.0, 91.8) | 91.8 (58.5, 100.0) |
| Urinary Irritative / Obstructive | 84.4 (75.0, 90.6) | 81.2 (68.8, 87.5) | 93.8 (87.5, 93.8) | 87.5 (81.2, 93.8) |
| Bowel | 95.8 (87.5, 100.0) | 95.8 (83.3, 95.8) | 95.8 (91.7, 100.0) | 97.9 (87.5, 100.0) |
| Sexual | 17.3 (8.3, 46.5) | 29.2 (4.2, 70.8) | 26.3 (16.7, 66.7) | 22.9 (12.5, 35.0) |
| Hormonal | 100.0 (90.0, 100.0) | 90.0 (80.0, 100.0) | 95.0 (90.0, 100.0) | 95.0 (85.0, 100.0) |
| FACT, mean (SD) ^a^ |  |  |  |  |
| Physical well-being | 24.9 (3.0) | 24.7 (3.5) | 24.6 (3.8) | 24.4 (2.7) |
| Social well-being | 19.2 (5.5) | 17.7 (6.5) | 19.2 (5.1) | 19.5 (7.2) |
| Emotional well-being | 20.4 (2.5) | 19.8 (3.5) | 19.7 (4.2) | 17.8 (4.3) |
| Functional well-being | 20.9 (5.5) | 21.2 (4.1) | 21.5 (5.1) | 19.6 (5.5) |
| FACT-Prostate, mean (SD) ^a^ | 118.7 (16.9) | 116.4 (16.3) | 120.5 (20.4) | 116.9 (18.1) |
| FACIT-Fatigue, mean (SD) ^a^ | 128.5 (19.8) | 127.7 (15.4) | 128.1 (22.3) | 125.1 (20.7) |
| EQ-5D-5L, mean (SD) ^a^ |  |  |  |  |
| EQ-5D index value | 0.9 (0.1) | 0.9 (0.1) | 0.9 (0.1) | 0.9 (0.1) |
| EQ VAS | 75.9 (16.0) | 85.3 (7.2) | 77.5 (17.1) | 76.9 (14.3) |
| Weight (kg), mean (SD) | 85.5 (12.4) | 85.9 (11.7) | 87.9 (15.0) | 85.0 (12.7) |
| BMI, mean (SD) | 26.8 (3.5) | 26.7 (2.9) | 27.9 (4.8) | 25.6 (0.7) |
| Smoking status, n (%) |  |  |  |  |
| Ever smoked | 8 (33.3%) | 12 (48.0%) | 8 (29.6%) | 4 (14.3%) |
| Never smoked | 9 (37.5%) | 5 (20.0%) | 14 (51.9%) | 14 (50.0%) |
| Missing | 7 (29.2%) | 8 (32.0%) | 5 (18.5%) | 10 (35.7%) |
| Alcohol units per week, n (%) |  |  |  |  |
| < 14 units | 8 (33.3%) | 7 (28.0%) | 10 (37.0%) | 6 (21.4%) |
| >= 14 units | 5 (20.8%) | 5 (20.0%) | 5 (18.5%) | 3 (10.7%) |
| Missing | 11 (45.8%) | 13 (52.0%) | 12 (44.4%) | 19 (67.9%) |
| Abbreviations: N, number; SD, standard deviation; IQR, interquartile range; BW, brisk walking; PA, physical activity; kg, kilograms; ; GLTPAQ, Godin-Shephard Leisure-time physical activity questionnaire; ICSmaleSF, International Continence Society male short form; EPIC-26, Expanded Prostate Cancer Index Composite – 26; FACT, Functional Assessment of Cancer Therapy; FACIT, Functional Assessment of Chronic Illness Therapy; EQ VAS, EQ visual analogue scale.  ^a^ Higher scores represent better outcomes.  ^b^ Higher scores represent worse outcomes.  ^C^ One unit = 10ml of pure alcohol. | | | | |

| **Supplementary table 5. Secondary outcomes at 12 months post-randomisation** | | | | |
| --- | --- | --- | --- | --- |
|  | Intervention | | | |
|  | Brisk Walking and Metformin | Brisk Walking | Metformin | Control |
|  | (n = 24) | (n = 25) | (n = 27) | (n = 28) |
| GSLTPAQ, n (%) |  |  |  |  |
| Active | 9 (37.5%) | 9 (36.0%) | 7 (25.9%) | 5 (17.9%) |
| Moderately active | 4 (16.7%) | 1 (4.0%) | 5 (18.5%) | 4 (14.3%) |
| Insufficiently active | 0 (0.0%) | 0 (0.0%) | 3 (11.1%) | 1 (3.6%) |
| Missing | 11 (45.8%) | 15 (60.0%) | 12 (44.4%) | 18 (64.3%) |
| GSLTPAQ - Frequency of activity (sweat), n (%) |  |  |  |  |
| Often | 3 (12.5%) | 5 (20.0%) | 5 (18.5%) | 0 (0.0%) |
| Sometimes | 8 (33.3%) | 9 (36.0%) | 6 (22.2%) | 15 (53.6%) |
| Never/rarely | 4 (16.7%) | 3 (12.0%) | 7 (25.9%) | 4 (14.3%) |
| Missing | 9 (37.5%) | 8 (32.0%) | 9 (33.3%) | 9 (32.1%) |
| Adapted stages of change questionnaire, n (%) |  |  |  |  |
| Pre-contemplation | 2 (8.3%) | 1 (4.0%) | 5 (18.5%) | 4 (14.3%) |
| Preparation | 1 (4.2%) | 0 (0.0%) | 1 (3.7%) | 0 (0.0%) |
| Action | 1 (4.2%) | 2 (8.0%) | 1 (3.7%) | 3 (10.7%) |
| Maintenance | 9 (37.5%) | 8 (32.0%) | 8 (29.6%) | 7 (25.0%) |
| Missing | 11 (45.8%) | 14 (56.0%) | 12 (44.4%) | 14 (50.0%) |
| Adapted theory of planned behaviour questionnaire, mean (SD) ^a^ |  |  |  |  |
| Attitude (good) | 5.9 (1.6) | 6.4 (1.1) | 5.9 (1.8) | 5.9 (1.8) |
| Attitude (10K steps, good) | 6.1 (1.6) | 6.6 (0.8) | 5.8 (2.1) | 6.4 (1.4) |
| Attitude (pleasant) | 4.8 (2.3) | 5.8 (1.6) | 5.4 (1.9) | 5.2 (1.8) |
| Attitude (10K steps, pleasant) | 4.6 (2.4) | 5.6 (1.9) | 5.4 (1.9) | 4.9 (2.3) |
| Perceived norm (injunctive) | 6.1 (1.6) | 6.3 (1.5) | 5.3 (2.1) | 5.1 (2.4) |
| Perceived norm (10K steps, injunctive) | 5.9 (1.6) | 6.6 (1.0) | 5.5 (2.0) | 4.6 (2.7) |
| Perceived norm (descriptive) | 5.0 (1.5) | 5.4 (1.6) | 5.1 (1.8) | 4.2 (1.8) |
| Perceived norm (10K steps, descriptive) | 5.2 (1.7) | 5.5 (1.6) | 5.6 (1.5) | 4.4 (1.8) |
| Perceived behavioural control (capacity) | 5.3 (2.0) | 5.5 (2.0) | 5.7 (2.0) | 4.3 (1.8) |
| Perceived behavioural control (10K steps, capacity) | 5.3 (2.0) | 5.9 (1.8) | 5.8 (2.0) | 4.4 (2.5) |
| Perceived behavioural control (autonomy) | 6.4 (1.1) | 6.6 (1.5) | 5.5 (2.1) | 6.2 (1.5) |
| Perceived behavioural control (10K steps, autonomy) | 6.3 (1.2) | 6.9 (0.3) | 6.3 (1.4) | 6.6 (0.9) |
| Intention (PA) | 4.6 (2.1) | 4.4 (2.4) | 3.9 (2.1) | 3.6 (1.7) |
| Intention (10K steps) | 4.5 (1.9) | 4.9 (2.4) | 4.4 (1.9) | 4.3 (2.4) |
| Past behaviour (PA) | 5.5 (2.1) | 5.2 (2.1) | 4.2 (2.3) | 4.5 (2.2) |
| Past behaviour (10K steps) | 6.2 (1.6) | 5.0 (2.1) | 5.3 (1.6) | 5.6 (2.2) |
| Profile of mood states, median (IQR) ^b^ |  |  |  |  |
| Tension, median (IQR) | 4.0 (2.0, 7.0) | 1.0 (1.0, 4.0) | 2.0 (1.0, 5.0) | 5.0 (3.0, 9.5) |
| Depression | 2.0 (0.0, 6.0) | 1.5 (0.5, 4.0) | 1.0 (0.0, 5.0) | 3.0 (1.0, 8.5) |
| Anger | 2.0 (0.0, 7.0) | 1.0 (0.0, 4.0) | 2.0 (1.0, 6.0) | 3.0 (1.0, 4.0) |
| Fatigue | 2.0 (2.0, 8.0) | 2.0 (1.0, 7.0) | 2.5 (1.0, 9.0) | 6.0 (1.5, 9.0) |
| Confusion | 3.0 (3.0, 7.0) | 3.0 (3.0, 5.0) | 2.0 (1.0, 5.0) | 4.0 (3.0, 8.0) |
| Vigour | 18.5 (14.0, 22.0) | 21.0 (14.0, 23.0) | 17.0 (12.0, 24.0) | 16.5 (16.0, 19.0) |
| Total mood disturbance | 23.5 (0.0, 41.0) | 31.0 (0.0, 38.0) | 28.0 (0.0, 41.0) | 25.5 (0.0, 44.5) |
| Benefit finding, mean (SD) ^a^ | 48.5 (16.2) | 49.9 (15.9) | 49.7 (15.2) | 51.2 (16.2) |
| ICSmaleSF, median (IQR) ^b^ |  |  |  |  |
| Voiding | 6.0 (2.0, 9.0) | 5.0 (2.0, 8.0) | 2.5 (1.0, 5.0) | 4.0 (2.0, 7.0) |
| Incontinence | 2.0 (0.0, 7.0) | 1.5 (0.0, 3.0) | 2.5 (0.5, 4.0) | 1.5 (0.0, 6.0) |
| EPIC-26, median (IQR) ^a^ |  |  |  |  |
| Urinary Incontinence | 77.2 (56.2, 100.0) | 79.2 (58.5, 100.0) | 85.5 (71.0, 93.8) | 80.2 (66.8, 91.8) |
| Urinary Irritative / Obstructive | 87.5 (75.0, 93.8) | 87.5 (81.2, 93.8) | 93.8 (87.5, 100.0) | 87.5 (81.2, 100.0) |
| Bowel | 95.8 (87.5, 100.0) | 100.0 (91.7, 100.0) | 95.8 (91.7, 100.0) | 100.0 (83.3, 100.0) |
| Sexual | 16.7 (5.5, 62.5) | 41.7 (12.5, 83.3) | 36.2 (16.7, 61.2) | 16.7 (4.2, 30.5) |
| Hormonal | 87.5 (85.0, 100.0) | 90.0 (75.0, 97.5) | 93.8 (85.0, 100.0) | 92.5 (85.0, 100.0) |
| FACT, mean (SD) ^a^ |  |  |  |  |
| Physical well-being | 24.4 (2.9) | 24.4 (3.9) | 25.8 (2.4) | 24.7 (3.8) |
| Social well-being | 19.5 (6.3) | 18.9 (6.1) | 19.3 (5.5) | 19.3 (5.8) |
| Emotional well-being | 19.7 (2.5) | 20.1 (3.1) | 20.5 (3.5) | 19.2 (3.8) |
| Functional well-being | 22.1 (3.5) | 21.9 (5.6) | 23.4 (4.2) | 20.5 (5.9) |
| FACT-Prostate | 120.5 (15.4) | 121.6 (23.1) | 127.3 (15.4) | 119.4 (18.9) |
| FACIT-Fatigue | 129.2 (15.1) | 129.2 (25.8) | 133.8 (17.6) | 125.3 (23.3) |
| EQ-5D-5L, mean (SD) |  |  |  |  |
| EQ-5D index value | 0.9 (0.1) | 0.9 (0.1) | 0.9 (0.1) | 0.9 (0.1) |
| EQ VAS | 76.7 (14.1) | 82.6 (11.2) | 82.0 (12.6) | 78.8 (10.8) |
| Smoking status, n (%) |  |  |  |  |
| Ever smoked | 7 (29.2%) | 10 (40.0%) | 7 (25.9%) | 5 (17.9%) |
| Never smoked | 7 (29.2%) | 7 (28.0%) | 11 (40.7%) | 14 (50.0%) |
| Missing | 10 (41.7%) | 8 (32.0%) | 9 (33.3%) | 9 (32.1%) |
| Alcohol units per week, n (%) |  |  |  |  |
| < 14 units | 0 (0.0%) | 3 (12.0%) | 3 (11.1%) | 2 (7.1%) |
| >= 14 units | 13 (54.2%) | 9 (36.0%) | 12 (44.4%) | 7 (25.0%) |
| Missing | 11 (45.8%) | 13 (52.0%) | 12 (44.4%) | 19 (67.9%) |
| Abbreviations: N, number; SD, standard deviation; IQR, interquartile range; BW, brisk walking; PA, physical activity; kg, kilograms; ; GLTPAQ, Godin-Shephard Leisure-time physical activity questionnaire; ICSmaleSF, International Continence Society male short form; EPIC-26, Expanded Prostate Cancer Index Composite – 26; FACT, Functional Assessment of Cancer Therapy; FACIT, Functional Assessment of Chronic Illness Therapy; EQ VAS, EQ visual analogue scale.  ^a^ Higher scores represent better outcomes.  ^b^ Higher scores represent worse outcomes.  ^C^ One unit = 10ml of pure alcohol. | | | | |
